# Maintaining Maternal and Child Health Services During the Ebola Outbreak: Experience from Pujehun, Sierra Leone

**DOI:** 10.1371/currents.outbreaks.d67aea257f572201f835772d7f188ba5

**Published:** 2016-06-02

**Authors:** GianLuca Quaglio, Damiano Pizzol, David Bome, Atiba Kebbie, Zainab Bangura, Vandi Massaquoi, Clara Frasson, Donata Dalla Riva, Giovanni Putoto

**Affiliations:** Directorate-General for Parliamentary Research Services (EPRS), European Parliament, Brussels, Belgium; Operational Research Unit, Doctors with Africa, Padova, Italy; Ministry of Health and Sanitation, Pujehun District, Freetown, Sierra Leone; Pujehun Hospital, Ministry of Health and Sanitation, Freetown, Sierra Leone; Pujehun Hospital, Ministry of Health and Sanitation, Freetown, Sierra Leone; Pujehun Hospital, Ministry of Health and Sanitation, Freetown, Sierra Leone; Doctors with Africa CUAMM, Freetown, Sierra Leone; Doctors with Africa CUAMM, Project Department, Padua, Italy; Doctors with Africa CUAMM, Operational Research Unit, Padova, Italy

## Abstract

**Background:**

During the Ebola outbreak the overall confidence of the population in the national health system declined in Sierra Leone, with a reduction in the use of health services. The objective of this study is to provide information on understanding of how Ebola impacted maternal and child health services in Sierra Leone. Data come from an operational setting which is representative of the communities affected by the outbreak.

**Methods:**

By integrating hospital registers and contact tracing form data with healthcare workers and local population interviews, the transmission chain was reconstructed. Data on the utilization of maternal and neonatal health services were collected from the local district’s Health Management Information System. The main measures put in place to control the Ebola epidemic were: the organization of a rapid response to the crisis by the local health authorities; triage, contact tracing and quarantine; isolation, clinical management and safe burials; training and community sensitization.

**Results:**

A total of 49 case patients were registered between July and November 2014 in the Pujehun district. Hospitalization rate was 89%. Overall, 74.3% of transmission events occurred between members of the same family, 17.9% in the community and 7.7% in hospital. The mean number of contacts investigated per case raised from 11.5 in July to 25 in September 2014. The 2014 admission trend in the pediatric ward shows a decrease after beginning of June: the reduction was almost significant in the period July-December (p 0.05). The admission in the maternity ward showed no statistical differences in comparison with the previous year (p 0.07). Also the number of deliveries appeared to be similar to the previous year, without significant variations (p 0.41).

**Conclusion:**

The Ebola outbreak reduced the number of patients at hospital level in Pujehun district. However, the activities undertaken to manage Ebola, reduced the spread of infection and the impact of the disease in mothers and children. A number of reasons which may explain these results are presented and discussed.

## Introduction

The Ebola epidemic has significantly affected maternal and neonatal health services, which has been attributed to the fear of infection, for both health care workers and patients^1^^,^^2^. Reports from West Africa described cases of patients requiring emergency obstetric care who travelled to multiple facilities in search of providers who would be willing to treat them^3^^,^^4^. Bulter described that "the top 3 differential diagnoses of antepartum bleeding had evolved from abortion, ectopic pregnancy, and disorders of the placenta to Ebola" 5. This situation was exacerbated by the limited numbers of health personnel, even before the outbreak, and by the high numbers of healthcare workers who died of Ebola Virus Diseases (EVD) in West Africa, of whom an estimated 30% worked in the field of maternal and child health^6^.

The challenge of maintaining maternal and child health services, across countries affected by Ebola has been previously reported^7^^,^^8^. Understanding of the ways in which the Ebola outbreak impacted maternal and child health services is still a real challenge due to the limited and uncertain data available^2^^,^^9^. At present there is insufficient information to understand which factors enabled certain districts to continue to provide some core services while others collapsed or struggled to respond to the crisis^2^^,^^9^.

During the Ebola outbreak the population’s overall trust in the national health system declined in Sierra Leone, with a reduction in the use of health services. For example, between May-September 2014, the number of women coming to health facilities for delivery declined by 27% in the country^10^. However, considerable, and not immediately clear, variations were also observed at district level,as showed by two HFs surveys conducted in Sierra Leone, the first in October 2014 and the second in March 2015^11^^,^^12^.

In relation to this, it is important to consider the geographical distribution of EVD cases across the country. The three provinces of Sierra Leone are divided into 14 districts (Figure 1). The number of confirmed cases (and deaths) varied by district as showed by the following figures: Bonthe, Pujehun 1-20; Moyamba, Bo, Kono, Tonkolili, Koinadugu; 21-100; Kambia, 101-500; Western Area Urban, Western Area Rural, Kenema, Kailahun, Port Loko and Bombali: 501-4000^13^.

The two HFs surveys showed that some districts that were heavily affected by the crisis registered a heavy impact on maternal and child health services, while other districts less or minimally affected by the outbreak showed similar reductions. Other districts moreover reported even an increase in maternal and child health services use^9^^,^^11^^,^^12^.For example, while Kambia, Port Loko and Bonthe showed large reductions in facility-based delivery (between 38-41%) the Pujehun district showed a decreased of 5%. Similar geographic variation was seen in reduction in antenatal are visits^9^.

The use of services was also strongly influenced by the rainy season (May to October), which impeded access to health services for a range of reasons^11^^,^^14^.Data on seasonal variations (October 2013-January 2014 vs October 2014-January 2015) at national level show a decrease for facility-based delivery by 7% and 14% for antenatal care visits. However at district level the situation is different: Western Area and Port Loko, heavily affected by the outbreak, showed the highest reductions in facility-based deliveries (33 and 17% respectively) and antenatal care visits (34 and 27% respectively). Bonthe, where there were fewer EVD cases showed a decrease in facility-based delivery by 15% and in antenatal care visits of 18% compared with the same period of the previous year. The Pujehun district, showed a low decrease in antenatal care visits (1%) and even an increase of facilities –based deliveries by 6%^9^. In short, across the country there were a range of responses.

Pujehun district is located in the south of the country (Figure 1), with a population of 335,574 people.

**Map of Sierra Leone highlighting the Pujehun district:**
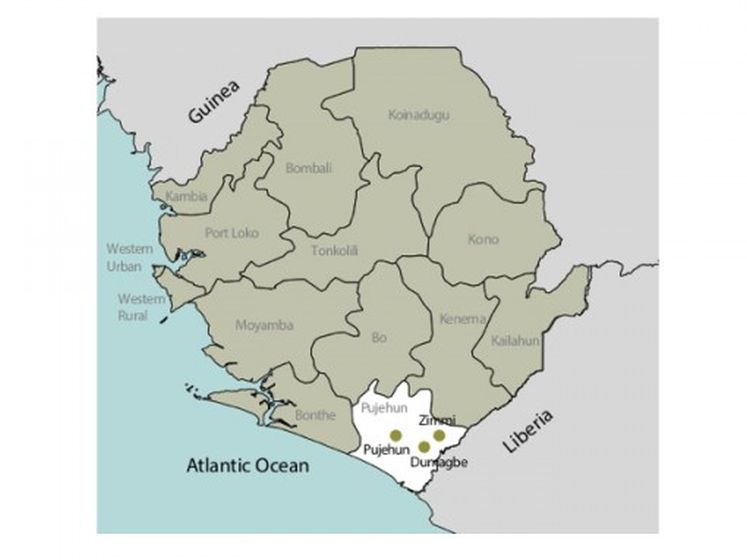

Connections within the district are made difficult because of the very poor status of the roads and the fact that the district is separated in two parts by a big river (Moa River). During the rainy season, access to the hospital is via barge, further complicating access. The majority of health services in Pujehun are provided by the Ministry of Health and Sanitation (MoHS). The primary care network includes 74 Peripheral Health Units (PHUs). The secondary care system consists of an 87 bed district hospital.

Doctors with Africa CUAMM (DwA) is an Italian NGO which has adopted the continuum of care approach as the main health service delivery strategy in its interventions^15^. In January 2012, DwA began to implement a three-year project funded by UNICEF in Pujehun district. In April-May 2014, many foreign aid agencies began to leave the areas affected by Ebola, as the situation was considered unpredictable^5^. When the first Ebola case in Sierra Leone was reported on May 23 2014, DwA decided to stay and to continue its work in the district as well as in the hospital.

The aim of this article is provide additional information on understanding of how Ebola impacted maternal and child health services in Pujehun district. The response to the Ebola crisis in Pujehun district is reported describing the organization of the activities implemented during the crisis. Data on the impact of Ebola outbreak on maternal and neonatal health care at primary level has already been shown by two HFs surveys conducted in Sierra Leone^11^^,^^12^. The contribution of this paper is to provide, general results on control interventions and data on the utilization of maternal and child hospital services in the district.

## Materials and Methods

**Organisation of the rapid response to Ebola**

As the epidemic developed in Sierra Leone in May 2014, the health authorities organized the health services strategy around the District Ebola Outbreak Control Plan and the Annual District Health Work Plan. Daily and weekly meetings were led by the District Health Management Team (DHMT) and the Hospital Management Team (HMT). The DHMT was supported by a number of national and international partners. These included DwA (Ebola case management, logistics, B-Emoc and C-Emoc services at hospital level); World Vision (child welfare, community engagement), Save the Children (contact tracing, infection, IPC), WHO (surveillance, IPC), UNFPA (safe burial), World Food Programme (supervision and supply of food) UNICEF (IPC, water and sanitation, children and orphans welfare) and local NGOs (community engagement).

Actions implemented during the EVD in Pujehun district (March 2014-Juanuary 2015) are summarised in Table 1.

**Figure:**
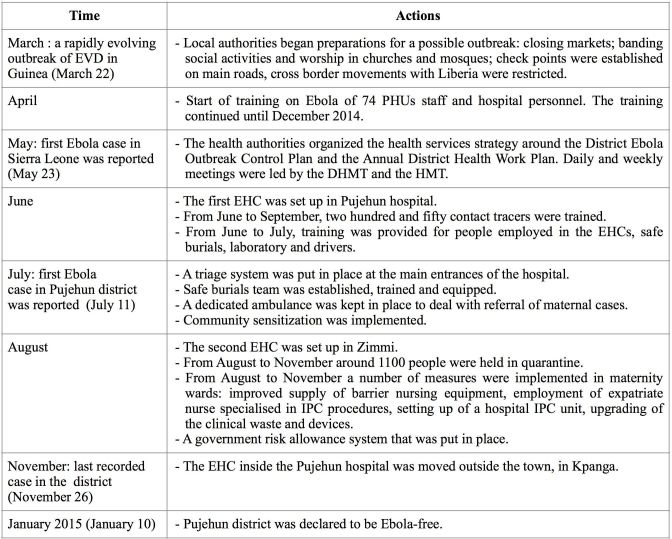
Table 1. Implemented actions during the Ebola crisis in Pujehun district, March 2014-Juanuary 2015

Following the reports of the outbreak in Guinea in March 2014, local authorities had begun to make preparations for a possible outbreak. These included a number of public health measures such as closing markets and banning social activities and worship in churches and mosques. Additionally, three check points were established on main roads, cross border movements with Liberia were restricted, bush meat sales were prohibited, and the transport of corpses was also prohibited.

**Isolation, clinical management and safe burials**

The Pujehun district recorded the first confirmed case of Ebola on 11 July, 2014, with the peak of the epidemic in September-October^16^. Two Ebola Holding Centres (EHCs) were set up: one in Pujehun hospital (end of June 2014, later moved outside the town in November), and one in Zimmi (August 2014), with a capacity of 20 beds overall. Patients with a positive result confirmed by laboratory testing were immediately transferred in 1-2 days to Ebola treatment centre in the neighboring districts. Unsafe burials were common at the beginning of the outbreak. In July, a safe burials team was established, trained and equipped.

**Triage, contact tracing and quarantine**

In July 2014 a triage system was put in place at the main entrances of the hospital. In the maternity complex mothers and children were first checked for Ebola by trained health workers. Based on clinical and epidemiological criteria (as indicated by MOH and WHO guidelines), suspected cases were referred to EHC for further investigation. From June to September 2014, about 250 contact tracers were trained and performed contact investigation. From August to November suspected cases were held in quarantine.

**Training, provision of personnel protective gear, infection prevention and control (IPC)**

The training on Ebola of 74 PHUs staff and hospital personnel started in April 2014, about 3 months before the first case was registered in the district. In June and July, further training was provided for people employed in the EHCs, safe burials, laboratory and drivers. Every month, staff in charge of PHUs met with the DHMT in Pujehun for continuous education, exchange of information and provision of available drugs and consumables. All the PHUs were initially provided with minimal protective equipment kit, and a number of personal protective equipment suits to be used in case of suspected Ebola cases. From September onwards, the supply of personnel protective equipment improved substantially.

In the maternity unit, normal deliveries and caesarean sections continued to be performed in the delivery room and operating theatre. At the beginning of the outbreak, poor infection control practice was a serious problem due to lack of protective equipment, inadequate hospital waste disposal, and staff behaviour. In order to address this, between August and November, a number of measures were implemented by the HMT such as: improved supply of barrier nursing equipment, employment of expatriate nurses specialized in IPC procedures, setting up of a hospital IPC unit, upgrading of the clinical waste and infrastructure such as a new incinerator. Throughout the period of the outbreak, a dedicated ambulance was kept to ensure the capacity to respond to requests to refer deliveries with maternal complications.

**Human resources**

The management of human resources during the Ebola outbreak was critical. Between May and December 2014 local health staff was strengthened with the presence of DwA international staff: on average during this period there was a team of additional expatriates on the ground (doctors and expert in public health, midwifes, nurses specialized in IPC in the maternity complex and logisticians). Continuing education was key for technical competency and staff motivation. Common decision making process for clinical and organizational issues androtating of staff proved were strategies adopted to reduce absenteeism, and to defuse tension and fear. The government risk allowance system that was put in place on August was an important incentive for staff motivation and retention.

**Community sensitization**

In Pujehun district, most of the Ebola cases appeared in the villages of Zimmi and Dumagbe. Initially, fear and mistrust were present in the affected communities as demonstrated by the fact that people who were suspected to have died from EVD were buried in secrecy. In Zimmi, local health personnel were threatened and had to leave. In response to this situation, the chief and community leaders were supported by the DHMT to encourage the population to collaborate with the international staff in implementing Ebola control measures. In general, throughout the district, awareness campaigns were developed during the Ebola outbreak with the aim of gaining the population's trust and encouraging them to seek medical care. These information, communication and educational activities were spread using different methods (radio messages, flip charts, flyers and posters, drama, door to door communication, religious functions, etc.). Measures taken during the Ebola outbreak aimed to target populations in a culturally sensitive manner, although there was limited evidence on what the most effective strategies were.

**Data collection**

Both confirmed and probable cases were considered. The transmission chain was reconstructed by analysing the registers of the two EHCs and contact tracing forms. In addition, healthcare workers involved in the management of the outbreak, survived case patients, and relatives of deceased case patients were interviewed. Data on hospital admission, and death/discharge were obtained from district health management team and registers of the two EHCs. Data on the utilization of maternal and neonatal health services were collected from the local district Health Management Information System, with the technical support of DwA.

**Statistical analysis**

Difference between data on maternal and neonatal health services were determined by two-tailed student’s t-test after acceptance of normal distribution with the Kolmogorov-Smirnov test. P values (two sided) of less than 0.05 were considered to be statistically significant. The DwA Ebola interventions were approved by the Ministry of Health, Sierra Leone. The study was approved by the Sierra Leone Ethics and Scientific Review Committee. Patient records/information was anonymized and de-identified prior to analysis.

## Results

**The outbreak and transmission chain**

A total of 49 case patients, consisting of 31 confirmed and 18 probable cases were registered between July and November 2014 in the Pujehun district. Of them, nine cases were imported into the district. The case fatality rate was 85.7% (42/49). Overall, 74.3% of transmission events occurred between members of the same household or extended family, 17.9% of transmission events occurred in the community (mainly between friends), and 7.7% of transmission events were healthcare related. Specifically, one driver was infected during the transport of two EVD cases from Zimmi to Pujehun EHC, one nursing aide was infected in the Pujehun EHC, and one patient was infected in Zimmi EHC.

Hospitalization, burials, contact tracing and training Between July and December 2014, around 60 people were admitted to EHCs in the Pujehun district. The number of patients hospitalized at the same time was never above 10. Among the 49 confirmed and probable cases patients, the percentage of unhospitalized cases was 11.2% (5/49). A total of 71.4% of fatal cases were buried the same day they died, with a maximum delay between death and burial of 2 days. About 250 contact tracers performed contact investigation. The mean number of contacts investigated for each Ebola case raised from 11.5 in July to 16 in August and reached 25 in September 2014. During the whole period of the outbreak, 1222 contacts listed were followed in the district. From August to November around 1100 people were held in quarantine. This number included 11 hospital health workers who had breached the safety procedures at the EHCs. From April to December, 41 days of training on Ebola were carried out. The majority of the health care workers in the region received one or more training.

**Pediatric admission, maternal admission, delivery**

Figure 2 shows the trends in admission in the pediatric ward in 2014, where a total of 750 patients were admitted (825 in 2013, p 0.23).

**Admissions to the pediatric ward of Pujehun hospital 2014, compared with data of 2013. Data are linked to Ebola cases.**
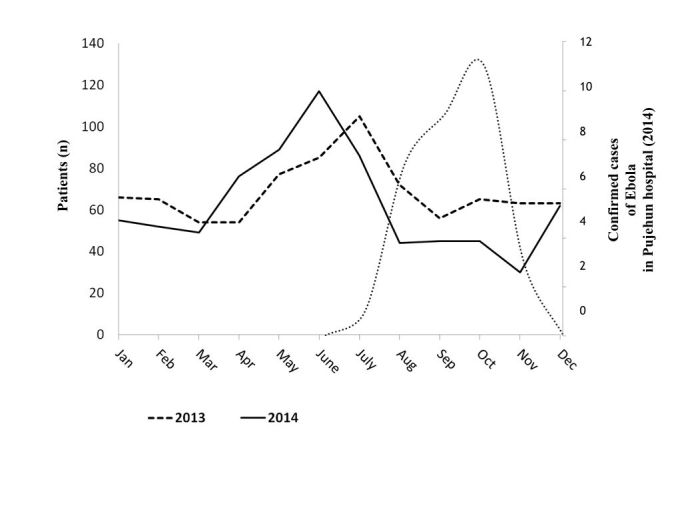

The 2014 trend shows an important decrease after the beginning of June, when the first Ebola case was reported in Sierra Leone. The total number of pediatric admissions in the period January-June 2014 was 438, not significantly higher than 2013 (401 in the same period: p 0.30). The reduction of pediatric admissions was almost significant in the period July-December going from 424 in 2013 to 312 in 2014 (p 0.05). In the period October-December 2014 very severe cases arrived to the hospital very late: therefore also the total death rate increased sharply to 13.3% (18 cases) in comparison to the 9% of the same period in 2013. Thirteen of 18 pediatric deaths (72%) happened in the first 24 hours, suggesting that the referrals from the PHUs were made with a critical delay. Figure 3 shows the maternity ward utilization.

**Admissions to the maternity ward of Pujehun hospital 2014, compared with data of 2013. Data are linked to Ebola cases.**
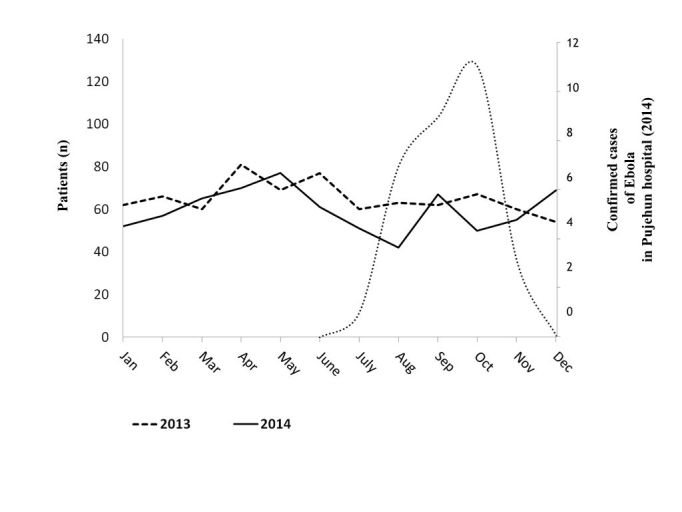

The admission in the maternity ward was 716 in 2014 (781 in 2013, p 0.07). The admissions in the maternity ward in the period January-June 2014 were 382 (compared with 425 in the same period in 2013: p 0.14). The maternity admissions in the period July-December 2014 were 334 (compared with 366 in the same period in 2013: p 0.13).

The total number of delivery in the Pujehun hospital was 460 in 2014 (453 in 2013, p 0.41) with a sensitive reduction between June-August 2014, before resuming a positive trend (Figure 4).

**Number of deliveries in Pujehun hospital in 2014, compared with data of 2013. Data are linked to Ebola cases.**
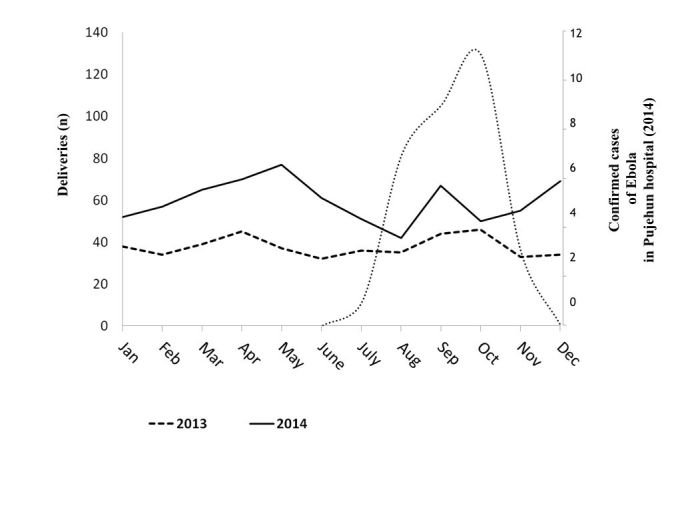

In the period January-June 2014 the deliveries were 226 (were 225 in the same period in 2013: p 0.48); in the period July-December 2014 were 234 (were 228 in the same period in 2013: p 0.41). In 2014, 124 caesarean sections were carried out (111 in 2013; p 0.15).

## Discussion

Effective containment of EVD in the Pujehun district is primarily ascribable to the intervention measures, such as an adequate number of EVD beds and the percentage of cases detected through contact investigation. These actions strongly depend on preparedness, population awareness, and compliance with intervention policies. Only one patient and two health care workers were infected in a hospital setting: this could perhaps reflect adequate healthcare system preparedness 17.

The Ebola outbreak was associated with a reduction in the number of patients attending PHUs at primary level (as showed by two HFs survey)^11^^,^^12^ and at hospital level in Pujehun district, as showed by this paper. However, the active role in managing the Ebola emergency in real time, reduced the spread of infection and the impact of the disease on routine care for mothers and children. In the maternity ward the number of admissions in October-December 2014 decreased but the number caesarean sections and the total deliveries increased. This suggests that the complicated deliveries were captured by the C-EmOC and that despite Ebola the trust of the people in the hospital capacity of safely managing obstetric emergency was maintained. There are a number of reasons which may explain these positive results in the Pujehun district: some related to the health services, some associated with other contextual factors.

**Factors related to the health services**

Pujehun was the first district in Sierra Leone to be given the all-clear after 42 days with zero recorded cases of the virus, and was declared the first Sierra Leone district Ebola-free on 10th, January 2015. One factor that may have contributed was the rapid response given by the district authorities in adopting important public health measures before any other district in Sierra Leone^18^. The health authorities kept the leadership in coordinating all the activities around the control of Ebola and the functioning of the maternal and neonatal health services. This was important since good governance is considered to be a key characteristic of a resilient health system^19^.

The activities were mainly concentrated on keeping the health service open and properly functioning in order to reduce the collateral effects of epidemics. No health units were closed during the epidemic. The supervision visits were focused particularly on the areas affected by Ebola hotspots (Zimmi and Dumagbe), while special attention was given to strengthening the referral system for obstetric cases. Special attention was paid to the implementation of IPC measures. The establishment up of a hospital IPC unit and the upgrade of the clinical waste and devices, contributed remarkably to improve the situation.

As described in previous experiences, health staff experienced severe emotional stress during the outbreak^20^^,^^21^. These arose from concerns over contracting Ebola, fears that they would infect their family, lack of motivation and tensions between and within the national and international teams were the main problems to face. Also the communication between patients and health workers were problematic. However, ensuring that staff had regular breaks and rotation, the provision of financial incentives and recognition were effective strategies to maintain teamwork and effective leadership.

As noted by Chowell and Nishiura (2014), “socio-cultural factors have not only contributed significantly to Ebola spread but have also complicated the implementation of control interventions”^22^. Gaining the trust of the population and community engagement was one of the biggest challenges in our experience^1^^,^^23^. Measures to empower community leaders and use culturally appropriate methods and channels of communication helped to dispel mistrust and misunderstanding in the communities. At the health unit level, a number of strategies and factors such as sharing information with the patients, providing answers to questions during care, being present in the daily activities of the hospital, acceptance of the professional risks, may have contributed to maintaining an attitude of ‘normality’ in an extremely stressful environment. We believe all this might explain population's positive receptiveness towards the hospital and the maternal and neonatal health services in general.

**Contextual factors**

In order to interpret these data correctly, it is important to consider a number of contextual factors which in our view enabled these activities in Pujehun district to continue.

First, as mentioned in the introduction, with the close district of Bonthe, Pujehun has been one of the districts least affected by Ebola in the country. This may be because it has one of the lowest population densities in the country. Residents live in villages of less than 2,000 people, and the capital, Pujehun town, has a population of just over 30,000. The outbreak affected a very isolated region and, in particular, two small, strictly connected areas, namely Zimmi (a rural town) and Dumagbe (a village).

Second, the hospital services were housed in two different buildings separated by few hundred meters: the first building hosts male and female wards and one of the two EHC were set up during the crisis; the second building (located 300 meters from the first) hosts maternity and pediatric wards. From the beginning of the Ebola epidemic this layout enabled checks for Ebola to be implemented at entrance of the pediatric and maternity complex.

Third, DwA was working in this community before the outbreak began, which gave an advantage of knowledge of the setting when the epidemic arrived which in turn facilitated mitigating measures to be put in place.

A fourth factor, which probably contributed to the reduction of the number of infections, was the rainy season. With no paved roads, the rainy seasons at its peak from August (when the first case was recorded in the district) make movement very difficult. While the rainy season (from May to October) may have reduced the access to health facilities, it may have helped to contain the spread of infection by making the road conditions difficult.

A fifth factor was the different timescale of the crisis in each district. Kailahun was the first district to report cases in May 2014, two months before the first cases in Pujehun. It is realistic to assume that public awareness for potential EVD cases was increasing across the country, and also in the Pujehun population, during that time^2^.

Finally, the Moa River separated the main hotspot of the epidemic (the surrounding area of Zimmi) from Pujehun town and the rest of the district, thereby creating a physical barrier.

Our experience raises a number of operational observations:

i) It is necessary to act as soon as possible in the situation of an epidemic, particularly in a context where the public health system is extremely fragile, adopting a holistic approach to get the whole picture of the situation in the field^24^;

ii) Early partnerships with local authorities and civil society leading to understanding their perspectives is also crucial, ensuring that the design and implementation of control measures are culturally appropriate^24^^,^^25^^,^^26^.

iii) As suggested by Iyengar et al.,^27^ it is necessary to address all aspects of healthcare that may be affected from the initiation of an emergency response, not just those related to the crisis, to reduce the negative impact on the maternal and neonatal care and maintain a population's acceptance towards the health services;

iv) The indirect consequences of the epidemic may be greater than the damage caused by the epidemic itself; therefore maternal and neonatal care in the Ebola outbreak and other emergency situation should receive special attention^6^^,^^7^;

v) Emergency and development need to be commonly investigated to strengthen the resilience of the local health system. The challenges of maintaining comprehensive maternal and neonatal health services during the Ebola outbreak is an example of the different issues surrounding the gap between emergency humanitarian aid and long term development aid^28^.

In conclusions, the strength of this study is that it uses data from a remote rural district setting in Sierra Leone during the Ebola outbreak. In the Pujehun district, even when faced with the extraordinary challenges of the Ebola outbreak, local leadership which implemented early response measures; continuity of care and staff protection; human resources management and community involvement were all important. We hope that these approaches can help others to continue their work in maternal and neonatal health care in challenging circumstances.

## Competing Interest

The authors have declared that no competing interests exist. The views expressed here are the sole responsibility of the authors and do not necessarily reflect the views of the affiliated organizations.

## Data availability

All data underlying the findings described in the manuscript are fully available without restriction.
